# High nuclear expression levels of histone-modifying enzymes LSD1, HDAC2 and SIRT1 in tumor cells correlate with decreased survival and increased relapse in breast cancer patients

**DOI:** 10.1186/1471-2407-14-604

**Published:** 2014-08-20

**Authors:** Remco S Derr, Anneke Q van Hoesel, Anne Benard, Inès J Goossens-Beumer, Anita Sajet, N Geeske Dekker-Ensink, Esther M de Kruijf, Esther Bastiaannet, Vincent THBM Smit, Cornelis JH van de Velde, Peter JK Kuppen

**Affiliations:** Department of Surgery, K6-R, Leiden University Medical Center (LUMC), P.O. Box 9600, 2300 RC Leiden, The Netherlands; Department of Pathology, LUMC, Leiden, The Netherlands

**Keywords:** Biomarkers, Breast cancer, Clinical outcome, Epigenetics, HDAC2, Histone-modifying enzymes, LSD1, SIRT1

## Abstract

**Background:**

Breast cancer is a heterogeneous disease with a highly variable clinical outcome in which both genetic and epigenetic changes have critical roles. We investigated tumor expression levels of histone-modifying enzymes LSD1, HDAC2 and SIRT1 in relation with patient survival and tumor relapse in a retrospective cohort of 460 breast cancer patients. Additionally, we correlated expression levels with tumor differentiation and tumor cell proliferation.

**Methods:**

Immunohistochemical staining for LSD1, HDAC2 and SIRT1 was performed on tissue microarrays of tumor and corresponding normal formalin-fixed paraffin-embedded tissues from breast cancer patients. Median nuclear expression levels in tumor tissues were used to divide the patients into low and high expression categories. In combined expression analyses, patients were divided into four subgroups: 1, all enzymes below-median; 2, one enzyme above-median; 3, two enzymes above-median; 4, all three enzymes above-median. The Cox proportional hazard model was used for univariate and multivariate survival analyses. The Pearson Chi-square method was used to assess correlation of combined expression levels with tumor cell proliferation and tumor differentiation.

**Results:**

Expression of LSD1 and SIRT1, but not of HDAC2, was significantly increased in tumor tissues compared to their normal counterparts (both p < 0.001). Multivariate survival analyses identified SIRT1 as independent prognostic factor for relapse-free survival (RFS) with a hazard ratio (HR) of 1.34 (95% CI = 1.04-1.74, p = 0.02). For overall survival (OS), no significant differences were found when the individual enzymes were analyzed. Analyses of combined expression levels of the three histone-modifying enzymes correlated with OS (p = 0.03) and RFS (p = 0.006) with a HR of respectively 1.49 (95% CI = 1.07-2.08) and 1.68 (95% CI = 1.16-2.44) in multivariate analyses and were also related to tumor differentiation (p < 0.001) and tumor cell proliferation (p = 0.002).

**Conclusions:**

When the combined expression levels were analyzed, high expression of LSD1, HDAC2 and SIRT1 showed shorter patient survival time and shorter time to tumor relapse and correlated with poor tumor differentiation and a high level of tumor cell proliferation. Expression of these histone-modifying enzymes might therefore be involved in breast cancer pathogenesis.

## Background

Clinical outcome of breast cancer patients is widely variable, due to the molecular heterogeneity of breast cancer. Breast cancer classification is based on a combination of several clinicopathological parameters, including histopathology, tumor stage, tumor grade and hormone receptor status and are used to guide treatment of breast cancer patients [1]. Even so, both over- and undertreatment of individual breast cancer patients occur, due to lack of reliable biomarkers [2, 3]. In order to further subclassify breast cancer patients, new prognostic biomarkers are warranted to improve the prognosis of individual breast cancer patients, based on their tumor characteristics. Such molecular biomarkers can be derived from biological mechanisms that underlie tumor growth and development.

Epigenetics is a rapidly developing field of research. Epigenetic mechanisms include DNA methylation, histone-modifying enzymes and their histone modifications. Due to the reversible nature of these processes, they are attractive targets for drug development and could be exploited to find novel prognostic biomarkers [3]. Histone-modifying enzymes are responsible for modification of certain residues on histone tails (histone modifications), thereby regulating DNA accessibility and expression of specific genes. Aberrant expression of histone-modifying enzymes, including lysine-specific demethylase 1 (LSD1), histone deacetylase 2 (HDAC2) and silent mating-type information regulation 2 homologue 1 (SIRT1), has been shown to have a role in breast cancer development [4–9] as well as prognostic value for breast cancer [10]. LSD1 is the first identified histone demethylase involved in specific demethylation of mono- and dimethylated lysine 4 on histone 3 (H3K4) and lysine 9 on histone 3 (H3K9) [4], and has been shown to increase with tumor progression [5]. HDAC2 is part of the class I HDACs and is responsible for deacetylation of histones and other protein targets [6]. Deacetylation of histones leads to compaction of the chromatin (heterochromatin) and reduced transcription of genes, including genes involved in processes such as cellular proliferation and cellular differentiation [6]. HDAC inhibition is currently investigated in clinical trials aiming to reverse hormone resistance in breast cancer [7]. SIRT1 deacetylates several histones and plays a role in tumorigenesis [8] and expression levels were increased in breast tumors compared to their matched normal breast tissues [9]. Recently, two publications showed that both histone demethylation inhibitors and histone deacetylation inhibitors, and especially a combination of the two agents, inhibit breast cancer cell growth *in vitro* [11, 12], suggesting an important role for histone demethylases and deacetylases in breast cancer.

LSD1, HDAC2 and SIRT1 are shown to act together in a single complex that represses transcription through compaction of the chromatin [13], thereby regulating gene expression. Therefore, we hypothesized that the combined expression levels of these collaborating histone-modifying enzymes in breast tumors is a stronger predictor for patient survival and tumor relapse than expression levels of the individual enzymes. Therefore, we investigated the correlation of the nuclear expression levels of LSD1, HDAC2 and SIRT1 as well as the combined expression levels of these enzymes with clinical outcome. The results showed that the expression levels of LSD1 and SIRT1 were increased in tumor tissues compared to adjacent normal breast tissues. Furthermore, overall survival (OS) and relapse-free survival (RFS) were decreased in breast cancer patients when tumor cells expressed high levels of all three markers. Finally, combined expression levels of the histone-modifying enzymes LSD1, HDAC2 and SIRT1 correlated with tumor differentiation and tumor cell proliferation.

## Methods

### Patient selection

The patient population was a retrospective cohort of female breast cancer patients (TNM: I-III) who underwent primary tumor resection at the Leiden University Medical Center (LUMC) between 1985 and 1996 (n = 822), as described previously [14]. Patients with bilateral tumors or a prior history of cancer (other than basal cell carcinoma or cervical carcinoma *in situ*) were excluded from the study. The following data were retrieved and used as covariates in multivariate analyses: age, tumor size, nodal status, expression of estrogen receptor (ER), progesterone receptor (PgR), human epidermal growth factor 2 (HER2), tumor grade, histological type, local and systemic therapy, survival time, and time until tumor relapse. All tumors were graded and histologically classified according to pathological standards by an experienced breast cancer pathologist (V.S.). The study was conducted with anonymized patient data according to Dutch law and in agreement with the Dutch Code of Conduct: “Proper Secondary Use of Human Tissue in the Netherlands” (Federation of Medical Scientific Societies, the Netherlands, http://www.federa.org/sites/default/files/bijlagen/coreon/codepropersecondaryuseofhumantissue1_0.pdf). The specific section is paragraph one of chapter eight on page 43 and therefore we did not ask for approval of an ethics committee [15], and according to the REMARK guidelines [16].

### Study design

Formalin-fixed paraffin-embedded (FFPE) tumor tissue of 701 patients, of whom tumor tissue was available, was included into a tissue microarray (TMA), as described previously [14]. For each patient, three cores of tumor tissue were included. For 261 breast cancer patients, of whom normal epithelial breast tissue was available, three cores of normal breast tissue were included in separate TMA blocks.

### Immunohistochemistry

TMA sections were cut (4 μm) and processed for immunohistochemistry (IHC). The antibodies that were used for IHC were validated by several other research groups: anti-LSD1 (ab17721, mouse, Abcam, Cambridge, United Kingdom) [17, 18], anti-HDAC2 (ab39669, rabbit, Abcam) and anti-SIRT1 (ab32441, rabbit, Abcam) [19]. The IHC was performed using a standard protocol [20]. Briefly, tissues were deparaffinized in xylene and rehydrated in a series of graded alcohol. Antigen retrieval was performed by heating the sections for 10 min in sodium-citrate buffer at 95°C (pH 6.0). Endogenous peroxidase activity was blocked with 0.3% hydrogen peroxide solution for 20 minutes. Incubation, with an optimized concentration of the antibodies described, was performed overnight at room temperature. Envision + peroxidase labelled polymer rabbit or mouse (Dako, Glostrup, Denmark) and DAB + liquid substrate chromogen system (Dako) were used for visualization of the expression levels. Counterstaining was performed using haematoxylin and dehydration was performed using graded alcohol and xylene.

### Evaluation of immunohistochemistry

The scoring of the immunohistochemical staining was performed by two investigators (A.S. and G.D.), who were blinded for the clinicopathological data. The percentage of positive stained tumor cell nuclei was scored in each of the tissue cores, from 0-100% with 10% increments. The second observer scored 30% of the tissue cores in order to determine consistency in quantification, which was tested with Cohen’s kappa coefficient for inter-observer variability. A Cohen’s kappa coefficient >0.6 was considered as substantial agreement. In addition to tumor tissues, stained normal epithelial breast tissue cores were also evaluated using the same scoring criteria as described above.

### Statistical analysis

Data were analyzed using SPSS 20.0 for Windows (SPSS Inc., Chicago, Illinois, United States of America). The paired student’s t-test was used to compare expression levels in tumor breast tissues and their corresponding normal epithelial tissues of 60 individual patients. The one-way ANOVA method was used for calculation of differences in expression levels between the TNM tumor stages (I-III) for LSD1, HDAC2 and SIRT1. For survival analyses, the patients were divided into a low and high expression category based on the median percentage positive tumor cell nuclei per enzyme. The Cox proportional hazards model was used for univariate and multivariate survival analyses. Kaplan-Meier (KM) curves and cumulative incidence curves were plotted to graphically show differences in patient survival and tumor relapse between the groups with different expression levels, respectively. For the uni- and multivariate analyses, only patients with nuclear staining data for all three enzymes and all covariates available, complete case analysis, were used in the statistical analyses (n = 460). Data were censored when patients were alive or free of relapse at their last follow-up date (lastly march 2013). Overall survival (OS) was defined as the time from date of surgery until death from any cause. Relapse-free survival (RFS) was defined as the time from surgery until the occurrence of a local, regional or distant tumor relapse or death by cancer. The Pearson Chi-square method was used to test for correlations between the combined expression levels of LSD1, HDAC2 and SIRT1 and clinical parameters. The low expression group was used as a reference in the single marker analyses. Low expression of all three markers was used as reference in the analyses of the combined expression levels. For the analyses of the combined expression levels of the markers, the patients were divided into four categories as follows: all enzymes below-median expression (‘all-low’), one enzyme above-median expression, two enzymes above-median expression and all three enzymes above-median expression (‘all-high’). We performed a Chi-square test between the four patients groups and all variables used as covariates, which are well-known independent prognostic factors in breast cancer and we corrected for those covariates in the multivariate analyses. For all analyses, a two-sided p-value ≤0.05 was considered statistically significant.

## Results

### Immunohistochemical staining of LSD1, HDAC2 and SIRT1 in breast tumors

**Figure 1:**
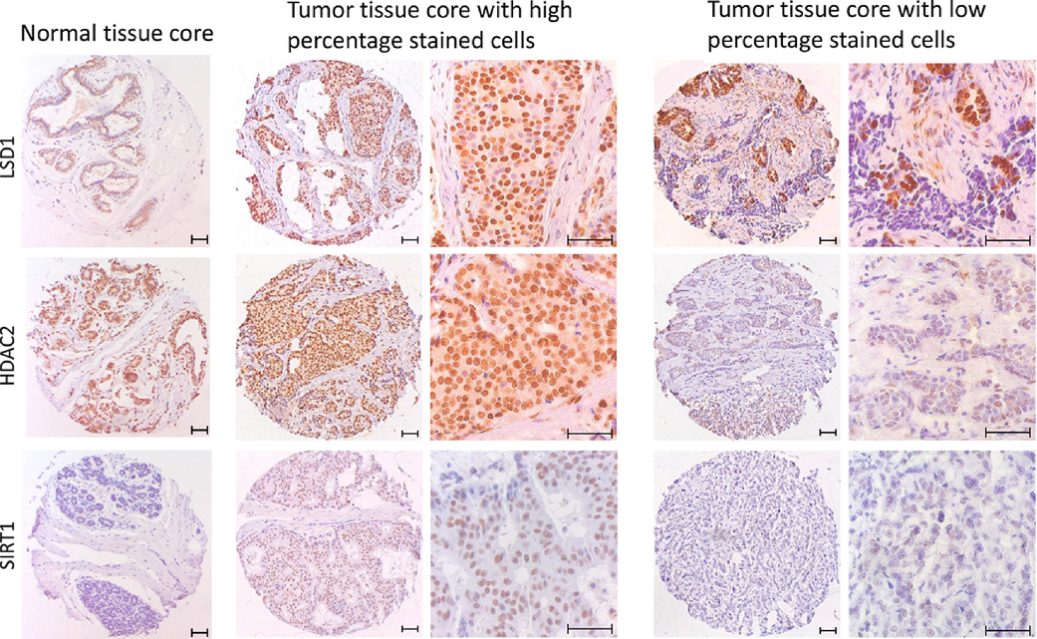
**LSD1, HDAC2 and SIRT1 expression in breast cancer.** Expression levels of LSD1, HDAC2 and SIRT1 were immunohistochemically determined in breast tumors as percentage of tumor cells with positive nuclear staining. The respective cut-off values, based on the median expression level, for low and high expression were 85% for LSD1, 80% for HDAC2 and 70% for SIRT1. For each staining a representative normal tissue core (left), a tumor tissue core with expression above median (middle) and below median (right) is shown. Pictures of the 0.6 mm tumor tissue cores were taken with a 100× magnification and a zoomed-in section of the tumor tissue cores is shown on the right of each image (400× magnification). The brown color represents the expression level of the enzymes.

**Figure 2:**
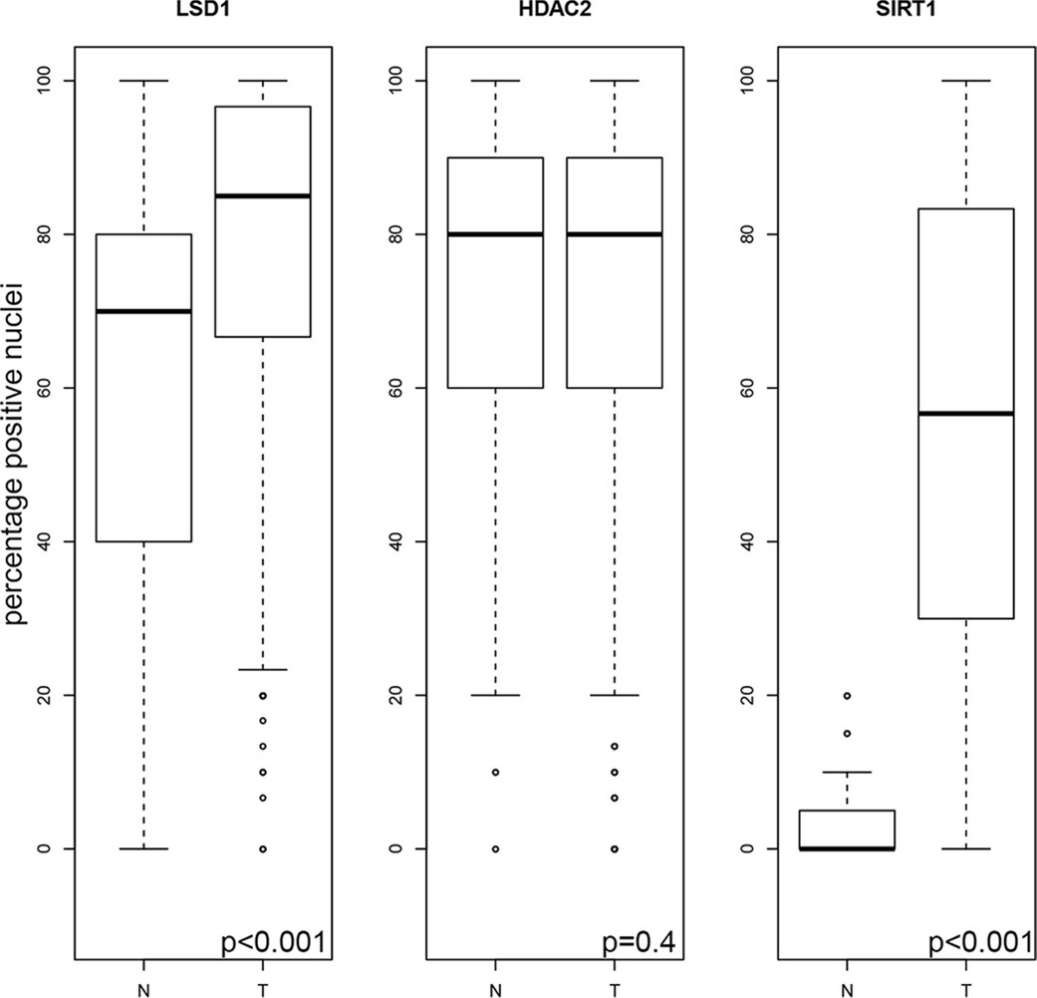
**SIRT1, HDAC2 and LSD1 expression in breast tumor tissues compared with normal epithelial breast tissues.** The boxplots show the mean percentage (horizontal line) of nuclei positive for LSD1, HDAC2 and SIRT1 in normal epithelial breast cells (labeled “N”) versus tumor breast cells (labeled “T”) for 60 patients with expression data of the histone-modifying enzymes for tumor tissues and normal epithelial tissues. Outliers are represented by circles. P-values were calculated using a paired student’s t-test and p-values ≤0.05 are considered as significant.

Table 1 shows the clinicopathological data of the breast cancer patients (n = 460) used for the statistical analyses of the three markers. The mean follow-up time was 11.8 years (range: 0.16-27.55 years) and the mean age at diagnosis was 58.3 years (range: 23–89 years). Percentages of positive nuclei for LSD1, HDAC2 and SIRT1 in the tumor and normal tissue cores were determined by IHC. Figure 1 shows representative pictures of normal breast tissue cores immunohistochemically stained individually for each enzyme, as well as representative pictures of breast cancer tissue cores with expression above and below median for each of the enzymes. The brown color is the amount of expression of the enzyme. The median percentages of positive tumor nuclei, used for the statistical analyses, were 85% for LSD1, 80% for HDAC2 and 70% for SIRT1. Cohen’s kappa coefficient was calculated to determine the inter-observer variability. The kappa coefficients for scoring of the tumor tissues were 0.664 for LSD1 and 0.627 for SIRT1. Both kappa coefficients were considered as substantial agreement between the observers. For staining of HDAC2 in tumor tissues, the kappa for scoring of the tumor tissue was not considered as substantial agreement. Therefore, a re-evaluation of the scoring was performed by the two observers until agreement was reached. For normal tissues the kappa coefficients were 0.693 for LSD1, 0.628 for HDAC2 and 0.605 for SIRT1, which were all considered as substantial agreement as well. The mean percentage of positive nuclei in the cores determined for each patient by the first observer, was used for survival analyses. Figure 2 shows the expression levels of LSD1, HDAC2 and SIRT1 in normal breast tissues compared to tumor tissues. Analyses of paired tumor and normal tissues showed an increased expression of LSD1 and SIRT1 in tumor tissues compared to normal tissues (both p < 0.001). HDAC2 expression did not significantly differ in tumor tissues compared to normal tissues (p = 0.4).

**Table 1:** **Clinicopathological data of the 460 breast cancer patients used in the study**

Characteristic	Mean (range)	N = 460	%
Follow-up (years)	11.8 (0.16-27.55)		
Age (years)	58.3 (23–89)		
<45		90	20
45-55		106	23
55-65		107	23
≥65		157	34
Tumor size (T)			
1		182	40
2		227	49
3-4		51	11
Nodal status (N)			
Negative		236	51
Positive		224	49
ER			
Positive		204	44
Negative		256	56
PgR			
Positive		223	49
Negative		237	51
HER2			
No overexpression		413	90
Overexpression		47	10
Histologic type			
Ductal		418	91
Other		42	9
Tumor grade			
1		71	16
2		222	48
3		167	36
Local treatment			
Mastectomy without RT		180	39
Mastectomy with RT		97	21
BCS with RT		183	40
Systemic treatment			
Chemotherapy alone		89	19
Endocrine therapy alone		77	17
Chemo- and endocrine therapy		18	4
None		276	60

Clinicopathological characteristics of the cohort of breast cancer patients. Statistical analyses were performed with all patients (n = 460) with complete clinicopathological data and nuclear staining data for LSD1, HDAC2 and SIRT1. Tumor size (T) and nodal status (N) were based on the TNM staging criteria. ER: estrogen receptor, PgR: progesterone receptor, HER2: human epidermal growth factor receptor 2, RT: radiotherapy, BCS: breast conserving surgery.

### Correlation of LSD1, HDAC2 and SIRT1 expression in tumor tissue with tumor stage

**Figure 3:**
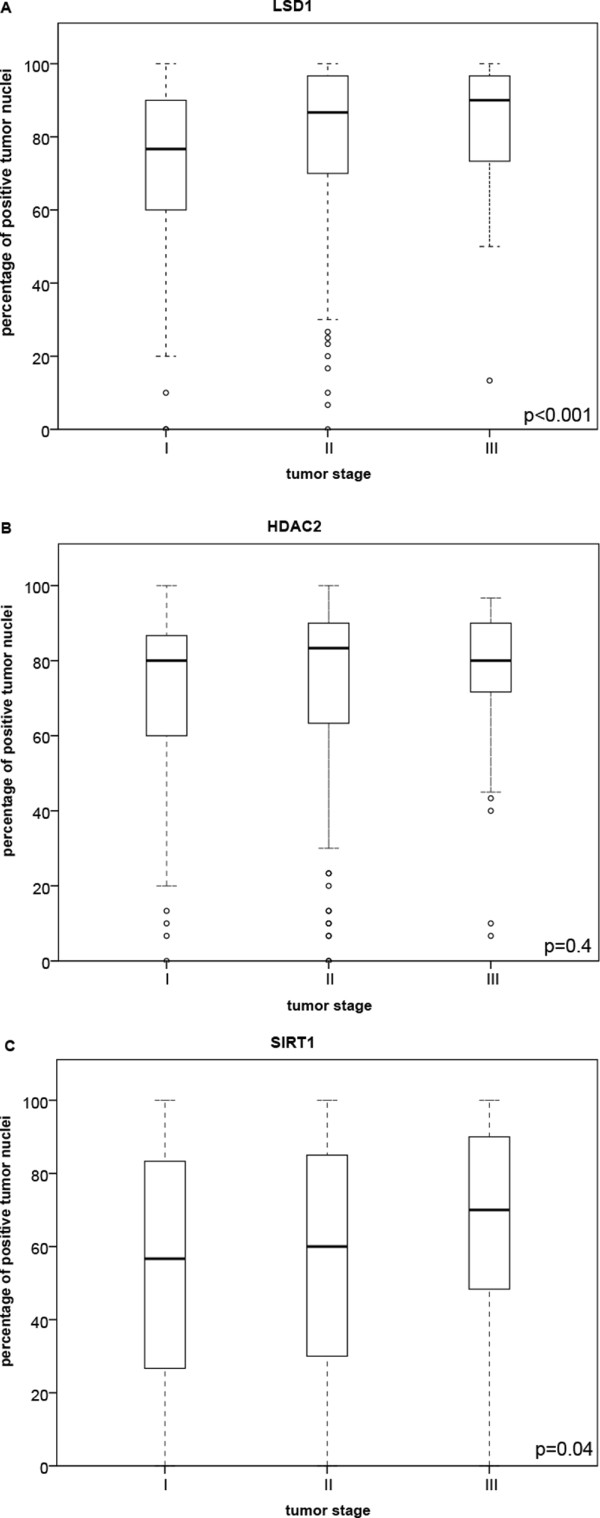
**Expression of LSD1, HDAC2 and SIRT1 in different tumor stages.** We included 182 patients with a stage I disease, 227 patients with a stage II disease and 51 patients with a stage III tumor. **(A)** Boxplot showing the percentage of positive tumor cells for LSD1 versus the TNM tumor stage (I-III) at moment of diagnosis. **(B)** HDAC2 expression levels versus TNM tumor stage shown in a boxplot. **(C)** The expression levels of SIRT1 versus TNM tumor stage represented in a boxplot. The thick horizontal lines represent the mean percentage of positive tumor cells in each category. Outliers are represented by circles. The p-values were calculated using the one-way ANOVA method and p-values ≤0.05 are considered as significant.

To investigate whether expression of each of the histone-modifying enzymes was related to the TNM tumor stage, the mean percentage of positive tumor nuclei was plotted against tumor stage. Figure 3 shows the percentage of positive nuclei in each tumor stage (I-III) for LSD1, HDAC2 and SIRT1. A one-way ANOVA analysis showed significant differences between the tumor stages for LSD1 (p < 0.001) and SIRT1 (p = 0.04) (Figures 3A and 3C). With higher expression in patients diagnosed with a higher tumor stage. HDAC2 did not show a significant difference between the tumor stages (p = 0.4) (Figure 3B).

### Prognostic value of single markers

**Figure 4:**
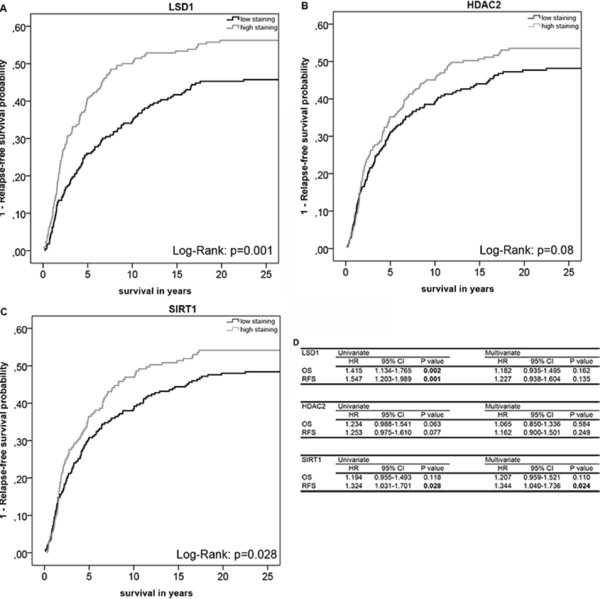
**Overall and relapse-free survival analyses of the expression levels LSD1, HDAC2 and SIRT1.** Cumulative incidence curves of the univariate relapse-free survival (RFS) analysis of LSD1 **(A)**, HDAC2 **(B)**, and SIRT1 **(C)** in breast tumors (n = 460). ‘Low expression’ was defined as expression level below median or equal to median and ‘high expression’ was defined as expression level above median. **(D)** Hazard ratios (HR), their 95% confidence intervals (95% CI) and their corresponding p-values for LSD1, HDAC2, and SIRT1 expression for overall survival (OS) and RFS were evaluated with the Cox proportional hazard model for uni- and multivariate analysis. Significant p-values (≤0.05) are indicated in bold.

Univariate analyses showed significant differences in patient survival and tumor relapse between patients with high and low nuclear expression of LSD1 (OS: p = 0.002, HR = 1.42, 95% CI = 1.13-1.77; RFS: p = 0.001, HR = 1.55, 95% CI = 1.20-1.99) and SIRT1 (RFS: p = 0.03, HR = 1.32, 95% CI = 1.03-1.70) (Figures 4A, 4C and 4D). No significant differences were observed for HDAC2 expression (OS: p = 0.1, HR = 1.23, 95% CI = 0.99-1.54; RFS: p = 0.1, HR = 1.25, 95% CI = 0.98-1.61) (Figure 4B and 4D). Multivariate analyses of the expression levels for individual markers showed a significant difference in RFS for SIRT1 (p = 0.02, HR = 1.34, 95% CI = 1.04-1.74) with shorter RFS in the high expression group (Figure 4D). No significant differences were observed for HDAC2 (OS: p = 0.6, HR = 1.07, 95% CI = 0.85-1.34; RFS: p = 0.2, HR = 1.16, 95% CI = 0.90-1.50) and LSD1 (OS: p = 0.2, HR = 1.18, 95% CI = 0.94-1.50; RFS: p = 0.1, HR = 1.23, 95%CI = 0.94-1.60) in the multivariate analyses (Figure 4D).

### Prognostic value of the markers combined

**Figure 5:**
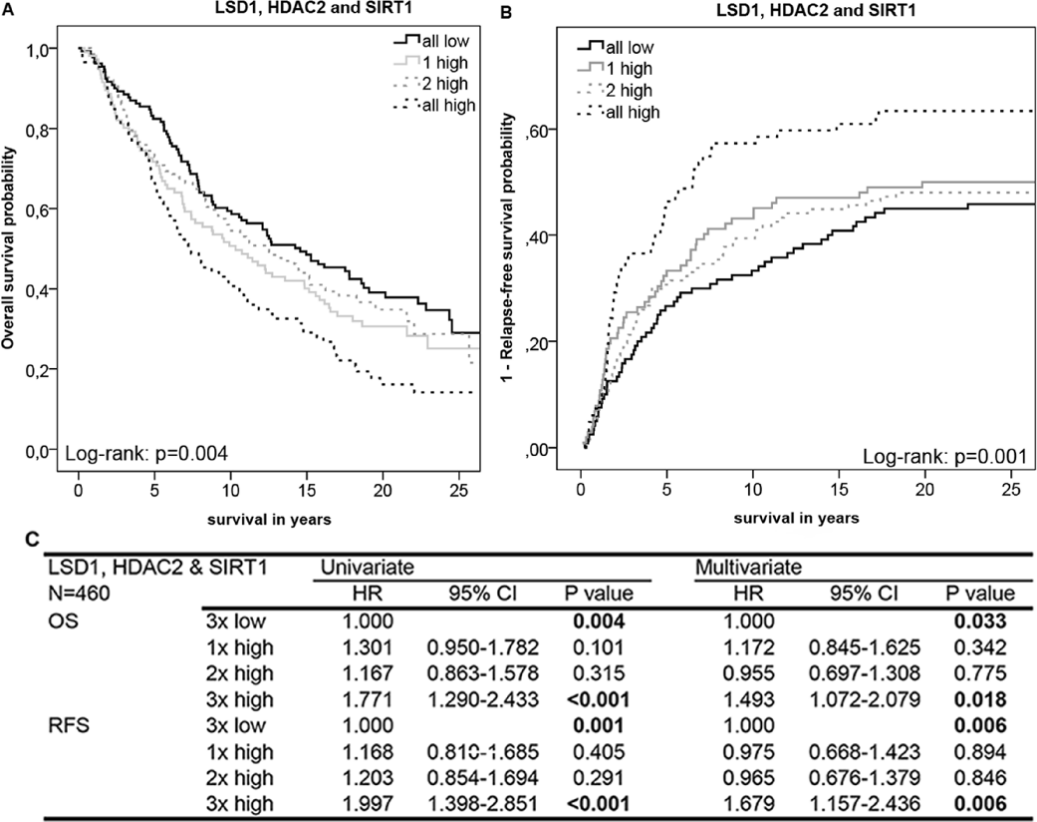
**Survival analyses of the combined expression levels of LSD1, HDAC2 and SIRT1.** Univariate Kaplan-Meier (KM) plot showing overall survival (OS) **(A)** and a cumulative incidence curve showing relapse-free survival (RFS) **(B)** of breast cancer patients for the combined expression levels of LSD1, HDAC2 and SIRT1. The patients were categorized in four subgroups depending on the expression levels of the histon-modifying enzymes. Subgroups: ‘All low’: expression of all three enzymes below median, ‘1 high’: one of the enzymes expressed above median, ‘2 high’: two enzymes expressed above median, ‘all high’: all three enzymes expressed above median. **(C)** The Cox proportional hazard model was used for evaluation of the HRs and the 95% confidence intervals (95% CI) of the combined expression levels of LSD1, HDAC2 and SIRT1 for OS and RFS in the four subgroups. Significant p-values (≤0.05) are indicated in bold.

Since the three enzymes work together in one complex, we hypothesized that the combined expression levels of the three histone-modifying enzymes is a stronger predictor for patient survival and tumor relapse than expression of individual enzymes. Survival analyses of OS and RFS showed that the combined expression level of LSD1, HDAC2 and SIRT1 in breast tumors was more predictive for patient survival and tumor relapse than each of the individual markers separately in both univariate and multivariate analyses (Figure 5). Chi-square analyses showed that there were significant differences between the four patient groups in ER (p = 0.019), PgR (p = 0.007), tumor grade (p < 0.001) and systemic therapy (p = 0.010), for which we corrected in the multivariate analyses. Multivariate analyses of the combined marker expression levels showed that patients with high expression level of all three markers had a shorter OS compared to patients with low expression of all the enzymes (p = 0.03, HR = 1.49, 95% CI = 1.07-2.08) (Figure 5C). For RFS the HR was 1.68 (p = 0.006, 95% CI = 1.16-2.44) in the ‘all-high’ expression group versus the ‘all-low’ expression group (Figure 5C). This result indicated that patients with high expression of all three enzymes have a shorter RFS compared to patients with one or more enzymes with a low expression level.

### Correlation of the combined expression levels of LSD1, HDAC2 and SIRT1 with tumor differentiation and tumor cell proliferation

We tested if there was a correlation between the combined expression levels of the three enzymes and tumor differentiation, a marker of aggressive tumors, in the whole study population. Indeed, a significant correlation between these expression levels and tumor differentiation was found (p < 0.001; Table 2). The results showed that 24% of the patients with low expression of all three enzymes had a well-differentiated tumor and only 12% of the patients with high expression of all three enzymes had a well-differentiated tumor. A low differentiation grade was found in 21% of patients with low expression of LSD1, HDAC2 and SIRT1 and 43% of the patients with high expression of all three enzymes had a low grade of tumor differentiation. In addition, we investigated the relation between the combined expression levels of LSD1, HDAC2 and SIRT1 and tumor cell proliferation, assessed by ki-67 expression, which is another marker of aggressive tumors. Ki-67 expression levels were determined by IHC previously in our study cohort [21] and data were available for 423 of 460 patients (92%). A significant correlation was found between the expression of ki-67 and the combined expression levels of the three enzymes (p = 0.002; Table 3). The results showed that in 68% of the patients with low expression of all three enzymes, there was no expression of ki-67, which indicated that there is only a low level of tumor cell proliferation in these patients. When at least one of the three histone-modifying enzymes showed high expression, we observed an increase in the percentage of ki-67 positive tumors (up to 56%), indicating more proliferation of the tumor cells in these patients. In summary, there are correlations between the combined expression levels of LSD1, HDAC2 and SIRT1 and tumor differentiation and between the combined expression levels of these enzymes and tumor cell proliferation.

**Table 2:** **Correlation between combined expression level of LSD1, HDAC2 and SIRT1 and tumor differentiation**

N = 460	3× low	1× high	2× high	3× high	Total
Tumor differentiation					
High	31 (23.7%)	9 (8.4%)	21 (15.4%)	10 (11.6%)	71 (15.4%)
Moderate	73 (55.7%)	52 (49.1%)	58 (42.3%)	39 (45.4%)	222 (48.3%)
Low	27 (20.6%)	45 (42.5%)	58 (42.3%)	37 (43.0%)	167 (36.3%)
Total	131	106	137	86	460
Chi-square: p < 0.001					

Tumor differentiation, according to tumor grade as assessed by an experienced pathologist, versus the combined expression levels of LSD1, HDAC2 and SIRT1 in 460 breast cancer patients are shown. Patients were divided in four subgroups based on the expression levels of the histone-modifying enzymes: all enzymes below median (3x low), one enzyme above median (1× high), two enzymes above median (2× high) and all three enzymes above median (3x high).

**Table 3:** **Correlation between combined expression level of LSD1, HDAC2 and SIRT1 and ki-67 expression**

N = 423	3× low	1× high	2× high	3× high	total
Ki-67					
No expression	81 (67.5%)	40 (44.0%)	67 (51.1%)	37 (45.7%)	225 (53.2%)
Expression	39 (32.5%)	51 (56.0%)	64 (48.9%)	44 (54.3%)	198 (46.8%)
Total	120	91	131	81	423
Chi-square: p = 0.002					

Ki-67 expression versus the combined expression levels of LSD1, HDAC2 and SIRT1 in 423 breast cancer patients are shown. Patients were divided into four categories based on the expression levels of the histone-modifying enzymes: all enzymes below median (3x low), one enzyme above median (1× high), two enzymes above median (2× high) and all three enzymes above median (3× high).

## Discussion

Our study identified combined expression levels of the histone-modifying enzymes LSD1, HDAC2 and SIRT1 as an independent prognostic factor for patient survival and tumor relapse in breast cancer patients. In addition, our results showed that the combined marker expression levels correlated with tumor differentiation and tumor cell proliferation. All these results implicated that high expression of all three enzymes is associated with a more aggressive phenotype of the breast tumors.

Histone-modifying enzymes are involved in numerous processes that are related to cancer, including cellular proliferation and differentiation [22]. There is increasing evidence that shows that aberrant expression of these enzymes has a role in (breast) cancer development and tumor growth [5, 6, 8, 9, 23]. LSD1 is overexpressed in various cancer types, such as bladder, lung and colorectal cancer [23]. In our breast cancer patient study cohort, an increase in the expression of LSD1 in tumor tissues was found compared with normal epithelial breast tissues. Our study also showed an increase in nuclear expression of LSD1 from tumor stage I to III, which has been described in literature by another group as well [5]. Furthermore, we demonstrated that SIRT1 expression levels were significantly increased in tumor tissues compared to normal epithelial breast tissues, which has also been described in literature [9]. The multivariate Cox proportional hazard analyses showed that SIRT1 expression was an independent prognostic factor for RFS, but not for OS in our breast cancer cohort, although a previous publication showed prognostic value for both [10]. This discrepancy can be explained by differences between patient cohorts, because our cohort contained older patients and we excluded patients with a TNM tumor stage IV disease from the study. In our cohort, HDAC2 expression was not significantly different in normal and tumor breast tissues and was not predictive for OS and RFS, confirming the results of the univariate OS analysis of Müller *et al.* [24].

Other groups have studied combinations of histone-modifying enzymes, but did not correlate these to clinical outcome. For example, Huang *et al.* showed *in vitro* that LSD1 and HDACs are involved in tumor cell proliferation, because synergistic inhibition of breast cancer cell proliferation was observed as compared to inhibition of the individual enzymes [11]. In the same study, microarray screening showed that inhibition of the enzymes led to reexpression of aberrantly silenced genes involved in processes such as cell differentiation and cell proliferation, which are frequently deregulated in breast cancer [11].

Our study is, to our knowledge, the first study that correlated the combined nuclear expression levels of these three histone-modifying enzymes with survival data in breast cancer patients. High expression of all three enzymes in tumor cells was correlated with reduced patient survival and shortened RFS compared to the expression level of the individual enzymes, implicating that LSD1, HDAC2 and SIRT1 act together in the same complex.

It has been shown in literature that all three histone-modifying enzymes, analyzed in our study, are individually involved in inhibition of functioning of p53 via direct modification of p53 (demethylation by LSD1 [25] and deacetylation by SIRT1 [26]) or inhibition of p53-DNA binding (HDAC2 [27]). p53 is a well-known tumor-suppressor and reduced functioning of p53 leads to reduced apoptosis, reduced cellular senescence and increased survival of cells with DNA-damage, due to reduced cell-cycle arrests, potentially leading to tumor development [25–27]. Therefore, we hypothesize that the complex of LSD1, HDAC2 and SIRT1 has important roles, next to chromatin repression, in regulating cell survival and that aberrant expression of this complex leads to sustained survival of tumor cells. Possibly, combined inhibition of multiple histone-modifying enzymes, such as LSD1, HDAC2 and SIRT1, could lead to improved treatment of breast cancer patients.

## Conclusions

In summary, we showed that the combined expression level of LSD1, HDAC2 and SIRT1 is a good predictor for OS and RFS in breast cancer patients. High expression of all three enzymes correlated with a more aggressive tumor phenotype, which makes this multi-enzyme complex an interesting target for breast cancer treatment. Future research for prognostic biomarkers should focus on analyses of such combinations of histone-modifying enzymes, acting together in multi-protein complexes, and their respective histone modifications. This can potentially further elucidate the complex epigenetic regulatory mechanisms in breast cancer, which will help identifying new targets for therapy.

## Abbreviations

BCS: Breast conserving surgery
CI: Confidence interval
ER: Estrogen receptor
FFPE: Formalin-fixed paraffin-embedded
H3K4: Lysine 4 on histone 3
H3K9: Lysine 9 on histone 3
HDAC2: Histone deacetylase 2
HER2: Human epidermal growth factor 2
HR: Hazard ratio
IHC: Immunohistochemistry
KM: Kaplan-Meier
LSD1: Lysine-specific demethylase 1
OS: Overall survival
PgR: Progesterone receptor
RFS: Relapse-free survival
RT: Radiotherapy
SIRT1: Silent mating-type information regulation 2 homologue 1
TMA: Tissue microarray.
